# Proteinuria-Lowering Effects of Proprotein Convertase Subtilisin/Kexin Type 9 Inhibitors in Chronic Kidney Disease Patients: A Real-World Multicentric Study

**DOI:** 10.3390/metabo11110760

**Published:** 2021-11-05

**Authors:** Patricia Muñoz Ramos, Yohana Gil Giraldo, Vicente Álvarez-Chiva, David Arroyo, Cristina Sango Merino, Francesc Moncho Francés, Javier Ocaña, Javier Reque, Emilio Sánchez-Álvarez, José Luis Górriz, Borja Quiroga

**Affiliations:** 1 Nephrology Department, Hospital Universitario de La Princesa, 28006 Madrid, Spain; patrizael@gmail.com (P.M.R.); yohagil@gmail.com (Y.G.G.); valvarezchiva@gmail.com (V.Á.-C.); 2 Nephrology Department, Hospital General Universitario Gregorio Marañón, 28007 Madrid, Spain; dvdrry@gmail.com; 3 Nephrology Department, Hospital de Cabueñes, 33394 Gijón, Spain; cristina.sango@gmail.com (C.S.M.); jesastur@hotmail.com (E.S.-Á.); 4 Nephrology Department, Hospital Clínico Universitario, INCLIVA, Universidad de Valencia, 46010 Valencia, Spain; francescmoncho@gmail.com (F.M.F.); jlgorriz@gmail.com (J.L.G.); 5 Nephrology Department, Fundación Hospital de Alcorcón, 28922 Madrid, Spain; jocana@fhalcorcon.es; 6 Nephrology Department, Hospital General de Castellón, 12004 Castelló de la Plana, Spain; javier.reque@hotmail.com

**Keywords:** CKD, kidney function, PCSK9i, proteinuria

## Abstract

Control of dyslipidemia in chronic kidney disease (CKD) is not always guaranteed with statins and/or ezetimibe. Proprotein convertase subtilisin/kexin type 9 inhibitors (PCSK9i) have opened up a new era in lipid control, but their effect on renal function and proteinuria in real life have not yet been evaluated. The aim of the present study was to analyze the evolution of renal function and proteinuria in a cohort of CKD patients treated with PCSK9i. This retrospective multicentric cohort study included CKD patients treated with PCSK9i. Baseline epidemiological data, comorbidities and laboratory findings (including estimated glomerular filtration rate [eGFR], proteinuria and lipid profile) were collected. The evolution of renal function, proteinuria and lipid profile was analyzed during the 1-year follow-up. The cohort included 76 patients (68% male, mean age 66 ± 10 years). The mean baseline creatinine was 1.55 ± 0.77 mg/dL, and the mean eGFR was 52 ± 22 mL/min/1.73 m^2^. Reductions in LDL-cholesterol, total cholesterol and triglycerides during the first month were 51 ± 25%, 32 ± 25% and 11 ± 40%, respectively, levels that remained stable throughout the first year (*p* < 0.001 for LDL-cholesterol and total cholesterol trends and *p* = 0.002 for triglyceride trend). During follow-up, proteinuria improved from 57 (9–481) to 30 (7–520) mg/g (*p* = 0.021). In addition, eGFR remained stable, and no adverse events were reported. In our cohort, dyslipidemia treatment with PCSK9i was associated with decreased proteinuria in CKD patients, an effect that might be due to reduced lipid nephrotoxicity. Clinical trials are needed to further investigate whether this impact on proteinuria can significantly slow CKD progression in the long term.

## 1. Introduction

Chronic kidney disease (CKD) is a leading cause of cardiovascular events, especially in advanced stages [1]. Both classic (hypertension, diabetes, dyslipidemia) and non-classic (uremia, anemia, mineral metabolism disorders) cardiovascular risk factors have an impact on prognosis in CKD patients [2].

Among classic factors, dyslipidemia is one of the least frequent treatment targets in CKD patients. Current recommendations for lipid management vary according to different guidelines. The latest dyslipidemia guidelines published by a nephrology society were the 2013 Kidney Disease Improving Global Outcomes (KDIGO) clinical practice guidelines [3], in which recommendations for treating dyslipidemia in CKD followed the “fire and forget” strategy, prescribing statin plus ezetimibe in all stage 3a-5 (estimated glomerular filtration rate [eGFR] < 60 mL/min/1.73 m^2^) CKD patients not on dialysis, independently of low-density lipoprotein cholesterol (LDL-C) levels and without further testing or dose adjustment. Contrary to these recommendations and based on more recent epidemiological studies, the latest European Society of Cardiology and European Atherosclerosis Society guidelines suggested a “the lower, the better” strategy [4]. These guidelines classified cardiovascular risk of CKD patients by renal function into high (eGFR between 60 and 30 mL/min/1.73m^2^) or very high cardiovascular risk (eGFR below 30 mL/min/1.73m^2^). LDL-C targets for these patients were recommended as below 70 and 55 mg/dL, respectively [5].

Currently available drugs for lowering LDL-C levels to recommended targets include statins, fibrates, ezetimibe and omega-3 fatty acids. However, most have proven insufficient for reaching the recommended goals, and they may present adverse reactions such as muscle or liver toxicity [6].

In this setting, a new pharmacological group has emerged, targeting liver-derived circulating proprotein convertase subtilisin/kexin type 9 (PCSK9) [7]. The inhibition of PCSK9 leads to substantial reductions in LDL-C levels and improved cardiovascular event-free survival, as revealed by the results from the pivotal FOURIER and ODYSSEY OUTCOMES randomized clinical trials (RCT) [8,9]. Although some sub-analyses have shown the importance of PCSK9 inhibitors (PCSK9i) for CKD patients in terms of LDL-C and cardiovascular event rate reduction, limited information has been published regarding the evolution of renal function and proteinuria with their use [10,11]. Furthermore, both studies lacked data on proteinuria/albuminuria, and they systematically excluded patients with advanced CKD (eGFR < 20 mL/min/1.73 m^2^) [10,11].

The relationship between lipids and kidney function is bidirectional. First, CKD patients present a dysregulated lipid profile [12,13], in which lipoproteins are altered in both quantity and composition, enhancing an altered atherogenic profile [14]. Several mechanisms can explain those changes, including a reduction in hepatic and tissue lipoprotein lipase activity and an increase in levels of the enzyme PCSK9, resulting in impaired LDL-C recycling in the liver. In addition, immature HDL-C in plasma is increased, LDL-C particles become more oxidized and apolipoproteins increase substantially [15,16]. Nephrotic syndrome has been associated with a particularly dysregulated lipid metabolism. In this respect, new observations have likened PCSK9 expression in the cortical collecting ducts of primary glomerulonephritis patients to hypercholesterolemia, proposing the kidney, as a source of PCSK9, as a first-line therapeutic target for dyslipidemia in this population [17,18]. Recent rat-based studies have also shown that proteinuria can induce hypersulfated hepatic heparan sulfate side chains of heparan sulfate proteoglycan, increasing the affinity to PCSK9 in sinusoids, and thus worsening dyslipidemia [19].

Second, dysregulated lipids can deposit in the kidney [20], causing high levels of very low-density lipoprotein cholesterol, intermediate-density lipoprotein cholesterol and oxidized LDL-C to accumulate in renal mesangial cells, leading to mesangial proliferation and ultimately glomerular sclerosis [15]. Furthermore, free fatty acids have a demonstrated ability to alter podocyte cytoskeleton structure, inducing podocyte damage after binding to albumin, thereby causing tubulointerstitial injury after filtration through the damaged podocyte [21].

Given the absence of real-world data on the subject, we conducted a multicentric observational study to analyze changes in renal function and proteinuria in CKD patients treated with PCSK9i.

## 2. Results

### 2.1. Baseline Characteristics

As illustrated in Table 1**,** the cohort included 76 patients; in total, 52 (68%) were male, and mean age was 66 ± 10 years. As regards comorbidities, 64 patients (84%) had hypertension and 52 (68%) were diabetic, while 15 patients (20%) had familial hypercholesterolemia. Turning to treatment, 68 (90%) patients were receiving statins prior to PCSK9i, 45 (59%) ezetimibe and 12 (16%) fibrates; 49 patients (65%) were receiving renin–angiotensin–aldosterone system (RAAS) inhibitors; and two (3%) selective sodium–glucose cotransporter 2 inhibitors (SGLT2i).

### 2.2. Baseline Kidney Function

The most frequent CKD etiology was hypertension (42%), followed by diabetic kidney disease (21%). The mean baseline creatinine was 1.55 ± 0.77 mg/dL, and the mean eGFR was 52 ± 22 mL/min/1.73 m^2^. In total, 57 (75%) patients had a urine protein-to-creatinine ratio (UPCR), with a baseline median of 57 (interquartile range 9–481 mg/g) (Table 1).

### 2.3. Baseline Lipid Profile

Prior to PCSK9i initiation, lipid profile values were: total cholesterol (TC) 235 ± 71 mg/dL, LDL-C 154 ± 50 mg/dL, high-density lipoprotein cholesterol (HDL-C) 51 ± 12 mg/dL and triglycerides 160 (127–254) mg/dL (Table 1). PCSK9i treatment was indicated for failure to achieve LDL-C goals in 39 patients (51%) and for statin intolerance in 37 (49%). In all, 65 patients (85%) received evolocumab, and 11 (15%) alirocumab. Among those receiving evolocumab, the dose was 140 mcg twice a month in 97% (and 420 mcg per month in 3%), and the eight patients on alirocumab were prescribed 150 mcg once a month.

### 2.4. Lipid and Kidney Outcomes

**Figure 1:**
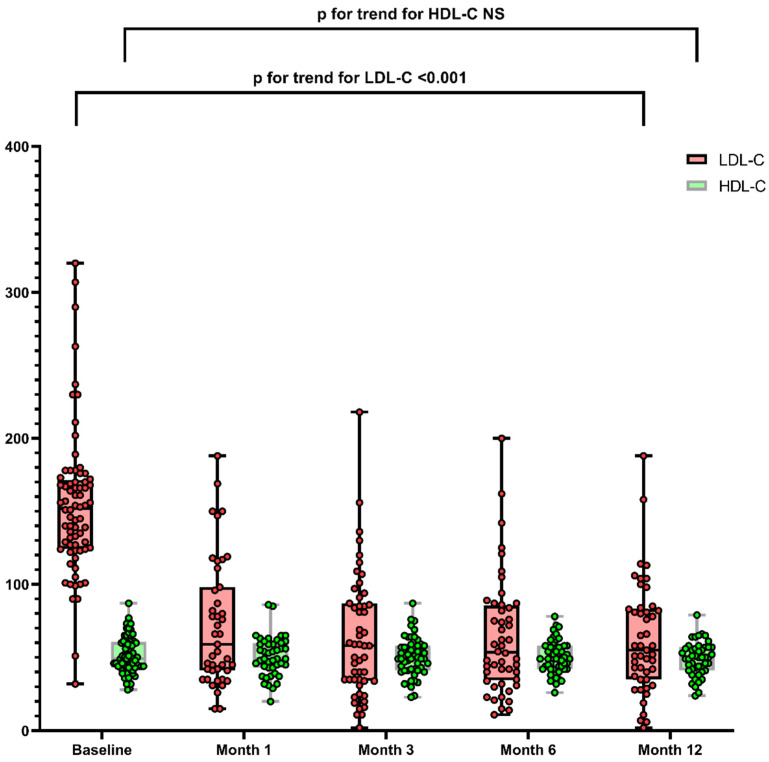
Evolution of LDL-C and HDL-C during follow-up of all included subjects. Values are expressed as median and interquartile range. Abbreviations: LDL-C: low-density lipoprotein cholesterol, HDL-C: high-density lipoprotein cholesterol, NS: non-significant.

The lipid profiles improved during follow-up. TC, LDL-C and triglycerides decreased after PCSK9i initiation and during the first year. Mean reductions at the 1-month follow-up were 51 ± 25% for LDL-C, 11 ± 40% for triglycerides and 32 ± 25% for TC. In contrast, HDL-C showed no significant changes (Table 2, Figure 1).

**Figure 2:**
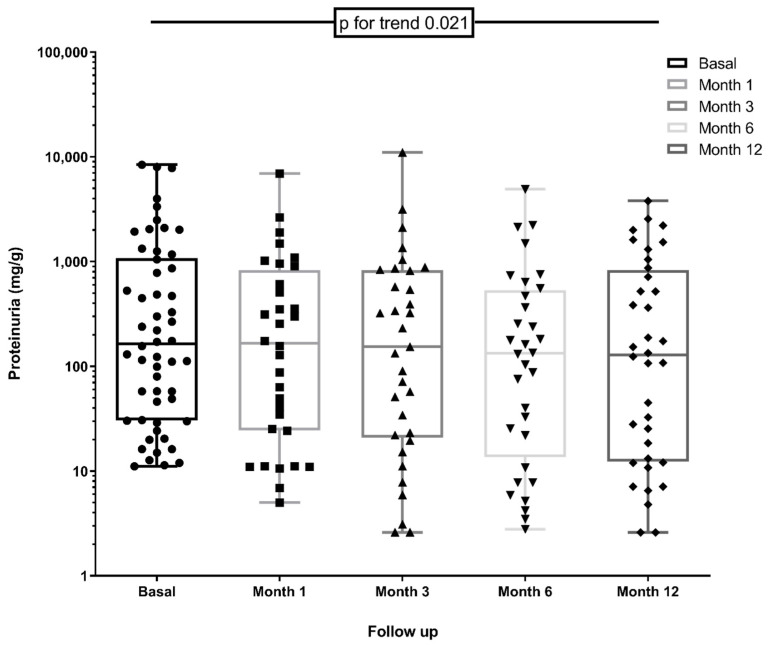
Changes in UPCR during follow-up from baseline (no PCKS9i treatment). Logarithmic plots showing median values and min/max range. Only patients with proteinuria > 10 mg/g (*n* = 57) are represented. Changes in proteinuria were assessed using linear models for repeated variables, showing a significant trend during 1-year follow-up.

**Figure 3:**
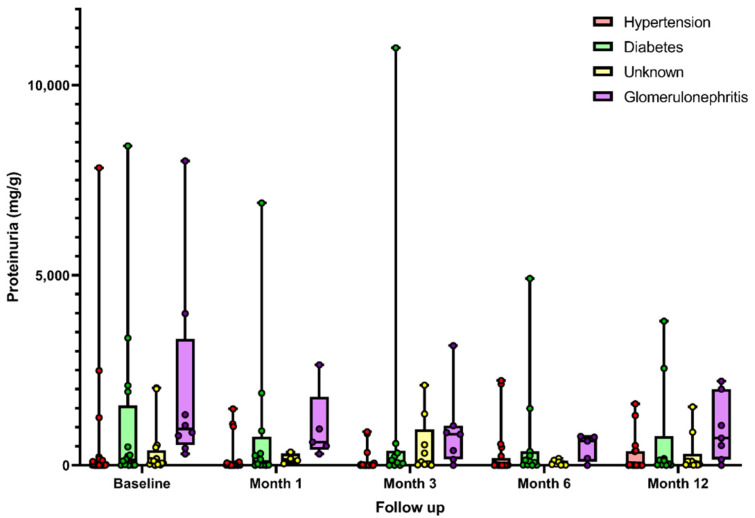
Descriptive plot of UPCR evolution according to the different etiologies of CKD. Values are expressed as mean and min/max range. Etiology was hypertension in 32 patients, diabetes in 16, unknown in 15 and glomerulonephritis in 7. No statistical significance for the trend was observed in subanalysis of each subgroup during follow-up (linear regression models). No adverse events were reported during the study.

As shown in Figure 2, the UPCR improved during the first month, a trend that continued through the 12-month follow-up (*p* for trend = 0.021). UPCR reduction rates compared with baseline were 20 (−3–42) % at 1 month, 32 (−2–64) % at 3 months, 41 (8–65) % at 6 months and 38 (9–64) % at the end of follow-up. We divided the sample to test whether proteinuria reduction was higher in any patient subsets. Interestingly, patients with baseline UPCR below the median (57 mg/g) achieved significantly higher reductions (*p* = 0.04). Figure 3 illustrates the evolution of UPCR by different CKD etiologies in a descriptive plot. As shown in Table 2, eGFR remained stable during follow-up.

## 3. Discussion

Our study demonstrates that dyslipidemia treatment with PSCK9i safely improves lipid profiles in CKD patients, with a concomitant decrease in proteinuria (as assessed by UPCR). This is a novel observation with these agents, which can be explained by the classic hypothesis known as “lipid nephrotoxicity” [22].

Our results invite the hypothesis that dyslipidemia control could decrease lipid-mediated damage in the kidney by the previously described mechanisms, which could explain the improvement in proteinuria seen in our series. This effect has previously been observed in treatment with statins, as shown in a meta-analysis by Palmer et al. [23], a study including 492 patients from nine RCTs in which, although data were heterogeneous, proteinuria decreased by 0.03–0.61 g/day on average. Although other studies have found no effect of statin therapy on proteinuria, there is a lack of well-designed clinical trials with the primary objective of assessing the impact of LDL-C on proteinuria [24,25]. To date, the effect of PCSK9i on proteinuria has not been published, but based on the toxic effects of lipids on the kidneys, it seems plausible that higher LDL-C reductions achieved with these drugs could favor more renal benefits, especially in terms of proteinuria. Unfortunately, albuminuria/proteinuria data were excluded from both the FOURIER and ODYSSEY studies. Nonetheless, the presence of proteinuria or even microalbuminuria (30–299 mg/g) is associated with an increased risk of cardiovascular disease and chronic kidney disease in diabetic and non-diabetic patients [26,27]. The measurement of albuminuria may predict kidney dysfunction and warrants renoprotective interventions. For this reason, the ESC recommends routine assessment of microalbuminuria in high- and very high-risk patients to identify those at risk of developing renal dysfunction and/or cardiovascular disease [28]. This should be taken into account in further studies on dyslipidemia. Albuminuria/proteinuria evaluation can provide valuable information on the possible additional benefits of lipid-lowering drugs in CKD patients at high or very high cardiovascular risk.

The effect of improving the lipid profile on the glomerular filtration rate is uncertain. Similar to our results, another meta-analysis conducted by Palmer et al. demonstrated no effect of statins use on eGRF [23]. As suggested by Nikolic et al. in their own meta-analysis, the beneficial effect of statins on renal function probably depends on the reduction of LDL-C levels, treatment duration and CKD etiology (given its differing prognosis) [25]. This time-dependent effect is of interest given that our follow-up reached 1 year, showing stabilization of eGFR and improvement of proteinuria. However, it remains to be demonstrated whether the mechanism for the possible beneficial effect of treating dyslipidemia on CKD progression is related to use of statins or directly conditioned by the decrease in LDL-C.

The beneficial effect of PCSK9i on proteinuria and glomerular filtration rate decline needs to be addressed. Evidence for a positive influence of PCSK9i on cardiovascular outcomes in CKD patients treated with statins has been published elsewhere [10]. Although the link between reduction in cardiovascular events and LDL-C has been demonstrated, proteinuria is now considered a major atherosclerotic risk factor [26,29]. Our results therefore lead us to theorize that PCSK9i exerts a double cardiovascular beneficial effect in proteinuric CKD patients, not only depending on LDL-C reduction, but also driven by the decrease of proteinuria promoted by the mitigation of lipid nephrotoxicity. Indeed, glomerulonephritis patients showed a greater (albeit non-significant) reduction in proteinuria. This can be explained by lipid damage recovery in glomerulus with PCSK9i administration, but also by inhibition of the newly described mechanism implicated in the dysregulation of dyslipidemia in proteinuric states [12,17,18]. Note that achieving the LDL-C target is likely to be even more difficult in nephrotic syndrome, which opens a clinical path to preferential PCSK9i prescription in this population [29].

PCSK9i has been widely demonstrated to reduce LDL-C in patients at a high risk of cardiovascular events [30]. This effect on lipid profile has also been achieved in CKD patients with both alirocumab and evolocumab in pivotal RCTs for both agents [10,11]. Our real-world data show a similar effect on LDL-C reduction (more than 50% at 1 month) but no effect on HDL-C levels, at least during the first year after treatment initiation. Similarly, a small clinical study including eight patients with refractory nephrotic syndrome and without history of cardiovascular events showed a safe reduction in LDL-C and TC (but not in HDL-C) [29]. HDL-C particles are dysfunctional but remain at normal levels in patients with nephrotic syndrome and tend to be low in CKD [31]. However, the impact on cardiovascular events of reducing HDL-C levels has not been clearly established in CKD, so targeting this lipoprotein is of lower priority than targeting LDL-C [32]. In our patients, baseline HDL-C was within the normal range, maybe due to the preventive measures (healthy diet, physical activity, statins) taken to reduce their high cardiovascular risk before prescribing PCSK9i. Specific studies based on CKD patients are required to analyze the cardiovascular effect via HDL-C increases.

The safety profile of PCSK9i in our study was excellent. Previous studies in CKD have shown only a slightly higher rate of injection-site reactions, without evidence of severe adverse effects after PCSK9i prescription [10,11]. Occasional case reports on tubular damage with PCSK9i have been published [33].

This study has some limitations; firstly, its retrospective design (due to which certain variables were missing, such as serum albumin and selective proteinuria) and the lack of a control group. As a real-world study, however, follow-up assays were available in most patients, ensuring a sufficient sample size to demonstrate the effect of PCSK9i on eGFR and proteinuria. Moreover, although we included patients with diverse CKD etiologies whose proteinuria might be affected by multiple factors, we limited this impact by excluding those who had received treatments with an effect on this parameter. Unfortunately, the sample size was not big enough for an in-depth analysis of the changes in proteinuria by CKD etiology; and finally, follow-up was probably not sufficiently long to detect significant variations in kidney function. Nevertheless, the beneficial effect of PCSK9i on proteinuria was significant and clinically relevant from the first month of follow-up.

## 4. Materials and Methods

### 4.1. Study Design and Patient Characteristics

We designed a multicenter retrospective cohort study. As the pilot center, we invited subjects to participate in the study via a memo sent to Spanish hospitals, finally including patients from five hospitals. Each center screened their CKD patients with PCSK9i, assessing criteria fulfilment and sending back information only on included subjects. Inclusion criteria were patients over 18 years with a CKD diagnosis according to KDIGO definitions [34] and an indication for treatment with PCSK9i, defined in the official therapeutic positioning report as an inability to achieve LDL-C < 100 mg/dL in patients with a previous cardiovascular event or with familial hypercholesterolemia, despite the maximum tolerated dose of statin and/or ezetimibe [35]. Exclusion criteria included a lack of treatment adherence, loss to follow-up during the first year, the development of concomitant kidney disease during the study (different from primary CKD etiology), changes in other treatments that might affect the assessment of kidney function or proteinuria (such as RAAS inhibitors, SGLT2i or immunosuppressive drugs), hospitalization and the need for renal replacement therapy. Patients with glomerulonephritis were included only if their primary kidney disease was stable, without any evidence of relapse. These criteria contributed to ensuring homogeneity in the sample.

We collected baseline demographic data and data on comorbidities (diabetes mellitus, hypertension, dyslipidemia, personal history of coronary disease, heart failure, stroke, peripheral vascular disease or familial hypercholesterolemia). Regarding CKD, baseline renal function was recorded as assessed by the CKD-EPI equation using creatinine and eGFR, UPCR and etiology [36]. CKD etiology was classified into hypertension, diabetic kidney disease, glomerular disease, hereditary kidney disease, tubulointerstitial kidney disease or unknown origin, following histopathological diagnosis if available or under the criteria of at least two nephrologists reviewing electronic health records.

Information was collected on other treatments, including statins, ezetimibe and fibrates; antiproteinuric drugs such as RAAS blockers or SGLT2i and immunosuppressive therapy. Lipid profiles included LDL-C, triglycerides, HDL-C and TC levels. Regarding PCSK9i treatment, prescription indication, PCSK9i type (evolocumab or alirocumab) and starting dose were also registered.

During follow-up, patients’ lipid and renal profiles were evaluated at 1, 3, 6 and 12 months after PCSK9i treatment initiation. The variation rates of lipids, eGFR and UPCR were calculated. UPCR variation was considered for analysis when it was higher than 10 mg/g at baseline. Adverse events were also collected.

This study was approved by the Research Ethics Committee of La Princesa University Hospital (Approval Number 04/20) and was conducted in accordance with the ethical standards of the Declaration of Helsinki (as revised in Brazil 2013).

### 4.2. Statistics

Values are expressed as mean ± standard deviation or median (interquartile range), depending on their distribution. Comparisons between variables were performed using chi-square or t-tests for parametric variables and Fisher or Mann–Whitney tests for non-parametric variables. Repeat measures of lipid and kidney parameters during follow-up (at 1, 3, 6 and 12 months) were evaluated using linear models for repeated variables, providing estimations of significance for each trend. An additional analysis was performed regarding CKD etiology.

All statistical analyses were performed with SPSS 24.0 (SPSS, Inc., Chicago, IL, USA). Figures were drawn with GraphPad Prism 6.0 (GraphPad Software Inc., San Diego, CA, USA). Results were considered statistically significant when *p*-values were <0.05.

## 5. Conclusions

In summary, treatment of dyslipidemia with PSCK9i in patients with non-dialysis CKD is associated with a reduction in proteinuria, which could partially be explained by the mitigation of lipid nephrotoxicity. Large clinical trials with strict endpoints focused on kidney function are warranted to address the real effect of PCSK9i on glomerular filtration rate and proteinuria. Reducing the atherogenic effect of dyslipidemia is mandatory in CKD patients, as they are at high cardiovascular risk even in the absence of previous vascular events.

## Figures and Tables

**Table 1:** Baseline characteristics.

Epidemiological Variables and Comorbidities	
Age (years)	66 ± 10
Sex (male, %)	52 (68)
Hypertension (*n*, %)	64 (84)
Diabetes mellitus (*n*, %)	52 (68)
Ischemic heart disease (*n*, %)	38 (50)
Heart failure (*n*, %)	17 (22)
Cerebrovascular disease (*n*, %)	12 (16)
Peripheral vascular disease (*n*, %)	14 (18)
**Treatment**	
Statins (*n*, %)	68 (90)
- Atorvastatin	46 (61)
- Rosuvastatin	13 (17)
- Others	9 (12)
Ezetimibe (*n*, %)	45 (59)
Fibrates (*n*, %)	12 (16)
RAAS inhibitors (*n*, %)	49 (65)
SGLT2i (*n*, %)	2 (3)
Immunosuppression (*n*, %)	0 (0)
**Kidney Function**	
Creatinine (mg/dL)	1.55 ± 0.77
eGFR (mL/min/1.73 m^2^)	52 ± 22
UPCR (mg/g)	57 (9–481)
CKD stages (*n*, %)	
- Stage 1	7 (9)
- Stage 2	23 (30)
- Stage 3a	16 (21)
- Stage 3b	18 (24)
- Stage 4	12 (16)
**CKD etiology (*n*, %)**	
- Hypertension	32 (42)
- Diabetic renal disease	16 (21)
- Unknown	15 (20)
- Glomerulonephritis	7 (9)
- Others	3 (4)
- Interstitial	2 (3)
- Hereditary	1(1)
**Lipid Profile**	
- Total cholesterol (mg/g)	235 ± 71
- LDL-C (mg/g)	154 ± 50
- HDL-C (mg/g)	51 ± 12
- Triglycerides (mg/g)	160 (127–254)

Values are expressed as mean ± standard deviation or median (interquartile range). Abbreviations: RAAS: renin–angiotensin–aldosterone system, SGLT2i: sodium–glucose cotransporter 2 inhibitors, CKD: chronic kidney disease, UPCR: urine protein-to-creatinine ratio, LDL-C: low-density lipoprotein cholesterol, HDL-C: high-density lipoprotein cholesterol.

**Table 2:** Evolution of lipid and renal profiles during follow-up.

	Baseline	1 Month	3 Months	6 Months	12 Months	*p* for Trend
Total cholesterol (mg/dL)	235 ± 71	156 ± 71	145 ± 58	139 ± 47	147 ± 41	<0.001
LDL-C (mg/dL)	154 ± 50	72 ± 43	64 ± 58	63 ± 39	62 ± 38	<0.001
HDL-C (mg/dL)	51 ± 12	50 ± 13	50 ± 13	50 ± 11	48 ± 11	0.583
Triglycerides (mg/dL) ^†^	160 (127–254)	138 (95–191)	130 (90–193)	130 (102–199)	146 (111–188)	0.002
Creatinine (mg/dL)	1.55 ± 0.77	1.58 ± 0.66	1.59 ± 0.61	1.55 ± 0.84	1.66 ± 0.91	0.737
eGFR (mL/min/1.73 m^2^)	52 ± 22	47 ± 20	46 ± 18	51 ± 21	48 ± 22	0.287
UPCR (mg/g) ^†^	57 (9–481)	34 (5–353)	22 (4–376)	25 (4–248)	30 (7–520)	0.021

Values are expressed as mean ± standard deviation or ^†^ median (interquartile range). Abbreviations: LDL-C: low-density lipoprotein cholesterol, HDL-C: high-density lipoprotein cholesterol, eGFR: estimated glomerular filtration rate, UPCR: urine protein-to-creatinine ratio.

## Data Availability

The data presented in this study are available on request from the corresponding author. The data are not publicly available to respect patient privacy.
